# Proteomics and phosphoproteomics of chordoma biopsies reveal alterations in multiple pathways and aberrant kinases activities

**DOI:** 10.3389/fonc.2022.941046

**Published:** 2022-09-30

**Authors:** Jing Hang, Hanqiang Ouyang, Feng Wei, Qihang Zhong, Wanqiong Yuan, Liang Jiang, Zhongjun Liu

**Affiliations:** ^1^ Center for Reproductive Medicine, Department of Obstetrics and Gynecology, Peking University Third Hospital, Beijing, China; ^2^ Key Laboratory of Assisted Reproduction, Ministry of Education, Beijing, China; ^3^ Beijing Key Laboratory of Reproductive Endocrinology and Assisted Reproduction, Beijing, China; ^4^ National Clinical Research Center for Obstetrics and Gynecology, Beijing, China; ^5^ Department of Orthopedics, Peking University Third Hospital, Beijing, China; ^6^ Beijing Key Laboratory of Spinal Disease, Beijing, China; ^7^ Engineering Research Center of Bone and Joint Precision Medicine, Beijing, China

**Keywords:** chordoma, proteomics, phosphoproteomics, integrated kinase network, kinase inhibitor

## Abstract

**Background:**

Chordoma is a slow-growing but malignant subtype of bone sarcoma with relatively high recurrence rates and high resistance to chemotherapy. It is urgent to understand the underlying regulatory networks to determine more effective potential targets. Phosphorylative regulation is currently regarded as playing a significant role in tumorigenesis, and the use of tyrosine kinase inhibitors in clinical practice has yielded new promise for the treatment of a variety of sarcoma types.

**Materials and methods:**

We performed comprehensive proteomic and phosphoproteomic analyses of chordoma using four-dimensional label-free liquid chromatography–tandem mass spectrometry (LC-MS/MS) and bioinformatics analysis. The potential aberrantly expressed kinases and their functions were validated using western blotting and CCK-8 assays.

**Results:**

Compared with paired normal muscle tissues, 1,139 differentially expressed proteins (DEPs) and 776 differentially phosphorylated proteins (DPPs) were identified in chordoma tumor tissues. The developmentally significant Wnt-signaling pathway and oxidative phosphorylation were aberrant in chordoma. Moreover, we predicted three kinases (AURA, CDK9, and MOK) with elevated activity by kinase-pathway network analysis (KiPNA) and verified their increased expression levels. The knockdown of these kinases markedly suppressed chordoma cell growth, and this was also the case for cells treated with the CDK9 inhibitor AZD4573. We additionally examined 208 proteins whose expression and phosphorylation levels were synergetically altered.

**Conclusions:**

We herein depicted the collective protein profiles of chordomas, providing insight into chordomagenesis and the potential development of new therapeutic targets.

## Introduction

Chordoma is a rare but malignant primary bone tumor that typically occurs along with the axial skeleton, with an incidence rate of approximately two cases per million people that represents 1%–4% of all bone cancers (1, 2). Chordoma is hypothesized to arise from the neoplastic transformation of embryonic notochordal remnant tissues (3). The rod-like notochord produces several signaling proteins (e.g., sonic hedgehog and Wnt) and secretes signaling factors (e.g., BMP and FGF) to direct organogenesis within the early embryo. The T-box-family transcription factor brachyury (*TBXT*), a crucial regulator of notochord development, and is characteristically overexpressed in chordomas and usually regarded as a hallmark of chordoma (4, 5). Despite its manifestation as a low-to-intermediate grade tumor that usually occurs sporadically, chordoma exhibits a strong tendency toward local recurrence, and over 30% of patients develop metastases (6).

As chordoma shows low sensitivity to conventional cytotoxic chemotherapies and radiation, radical surgery remains the primary and most effective treatment; however, their proximity to neurovascular structures hampers complete resection, and some patients have already developed metastatic diseases upon initial diagnosis. Several recent studies (including our own) have revealed that EGFR-signaling dysbiosis modulated by CMTM3 and cMET exacerbated the chordoma process (7, 8), and that DEPDC1B regulated ubiquitination of BIRC5 also promoted chordoma progression (9). However, more information should be depicted to overcome its lengthy latency and poor response to treatment (10).

Phosphorylation constitutes one of the most common post-translational modifications (PTMs) of proteins, with kinases exhibiting tumorigenic actions in various malignancies, and relevant small-molecule inhibitors have been widely and successfully employed for the clinical treatment of diverse types of cancer (11). Indeed, a series of tyrosine kinase inhibitors (TKIs) have been used in the pre-clinical trials of chordoma (12), including the inhibitors against the epidermal growth factor receptor (EGFR) like afatinib, erlotinib, and lapatinib (13), the inhibitors against tyrosine-protein kinase Met (c-MET) like crizotinib, the inhibitors against Platelet-derived growth factor receptor (PDGFR) such as imatinib (14), and the inhibitors of the type I IGF receptor/insulin receptor (IGF1R/INSR) inhibitor linsitinib together with erlotinib (15). However, their clinical applications all exhibited varied degrees of limitations. The responses of lapatinib were not as good as it could have been expected (16). Erlotinib incompletely suppressed chordoma growth *in vivo* (17). Not all PDGFR-positive chordomas paitients are sensitive to imatinib and it did not achieve dimensional and long-lasting responses as well (18, 19). Although the European multi-center clinical trial involving afatinib is still ongoing, it is well established that single-afatinib therapies have not yielded lasting effects and the tumoral evolutions lead to the drug resistance (20, 21). Besides, machine learning together with synergistic drug combinations have also been proposed in chordoma (22).

A majority of recent studies on chordoma have focused on histologic and genetic analysis (23–28). Moreover, although numerous investigators have assessed the roles of several individual proteins in chordoma (29, 30), we only uncovered a few reports on chordoma-related proteins that exploited proteomics (31–33) and little is known with respect to the PTMs of proteins. Therefore, a more comprehensive understanding of chordoma is urgently needed to elucidate phosphorylation status and the aberrant activation of signaling pathways that contribute to chordomagenesis.

In the present study, we profiled and integrated the proteomic and phosphoproteomic landscapes with label-free mass spectrometry, identified several kinases as potentially druggable molecular targets, and verified the effects of these kinases on chordoma cell proliferation. Our findings will enable us to better understand the underlying pathogenesis of chordoma, and thus yield novel targets and enhance the effectiveness of clinical treatments.

## Materials and methods

### Patients and tissue samples

Patients that had been diagnosed and treated as chordoma according to the preoperative images (including plain and enhanced head Magnetic Resonance Imaging (MRI), thin layer skull base Computed Tomography (CT) scanning, and 3-D reconstruction) at the Peking University Third Hospital were included in this study. All the cases were diagnosed by two independent pathologists using immunohistochemistry and histopathological examination. The detailed clinical characteristics of the enrolled fifteen patients are listed in **Table 1**. Chordoma tumor samples were obtained at the time of surgery, washed efficiently using ice-cold phosphate-buffered solution (PBS), snap-frozen in liquid nitrogen for 3 min, and stored at -80°C for further use. The distal normal muscle tissues were obtained as control and processed in the same way as chordoma samples. These paired tissues were used for the following integrated proteomics and phosphoproteomics analyses.

**Table 1:** Summary of clinical information of chordoma tumor samples and classification.

Patient Code	Age	Gender	Tumor size (cm)	Size classification ^#^	Site	primary or recurrent	usage in this study
C1	34	Male	3.8 × 4.8 × 5.4	Large	C	primary	Proteomics
C2	64	Male	2.8 × 4.4 × 3.4	Small	C	primary	Proteomics
C3	62	Male	3.4 × 3.7 × 5.8	Large	C	primary	Proteomics
C4	21	Female	5.9 × 4.4 × 7.5	Large	C	recurrent	Proteomics
C5	50	Female	2.5 × 2.0 × 2.8	Small	C	recurrent	Proteomics
C6	69	Female	2.4 × 1.5 × 2.4	Small	C	primary	Validation
C7	71	Female	4.3 × 4.2 × 3.8	Small	T	primary	Validation
C8	66	Female	2.4 × 2.7 × 3.1	Small	T	primary	Proteomics
C9	48	Male	9.7 × 2.3 × 2.6	Large	C	primary	Proteomics
C10	56	Female	3.7 × 2.6 × 2.3	Small	T	recurrent	Validation
C11	70	Male	14.6 × 9.8 × 14.3	Large	C	recurrent	Proteomics
C12	58	Male	3.1 × 2.1 × 4.8	Small	C	recurrent	Proteomics
C13	65	Female	9.8 × 3.4 × 4.8	Large	T	recurrent	Validation
C14	58	Female	3.9 × 4.2 × 5.1	Large	C	primary	Validation
C15	63	Male	3.1 × 2.2 × 2.7	Small	T	primary	Validation

^#^Large is identified as at least one edge is over 5.0 cm. C, cervical spine; T, thoracic vertebra.

### Sample preparation

#### Protein extraction and digestion

We weighed and ground samples into cell powder using a mortar and liquid nitrogen. Then we added four volumes of lysis buffer (8M Urea, 100 mM NaCl, 100 mM Tris-HCl, pH 8.0, 1% Protease Inhibitor, 1% Phosphatase Inhibitor) and treated the samples with sonication. The cell debris was removed and the supernatant was collected. The total protein concentration was determined with the BCA kit (Thermo Fisher Scientific, USA) following the manufacturer’s instructions.

The protein quantity and volume from each sample were adjusted to the same, and TCA were added to a final concentration of 20% and the proteins were precipitated at 4°C for 2 hours. The protein pellets were collected by centrifugation at 4,500 g for 5 min and washed with pre-cold acetone 3 times. 100 mM TEAB was added and the precipitation was dispersed by ultrasound. A 1:50 mass ratio of trypsin was used for the overnight digestion for 800 ug proteins. Then the solutions were reduced with 5 mM dithiothreitol (DTT) for 30 min at 56°C and alkylated with 11 mM iodoacetamide (IAA) for 15 min at room temperature in darkness. The digested peptides were desalted with C18 SPE (3M company) according to the manufacturer’s instructions.

#### Phosphopeptides enrichment

0.8 mg peptides per sample was used for phosphoproteomic analysis. Peptide mixtures were reconstituted in loading buffer (50% acetonitrile/6% trifluoroacetic acid) and enriched by immobilized metal-chelated affinity chromatography (IMAC) microsphere (High-Select™ Fe-NTA Phosphopeptide Enrichment Kit). After gentle incubation by rotation and successive washing with 50% acetonitrile/6% trifluoroacetic acid and 30% acetonitrile/0.1% trifluoroacetic acid, the bound phosphopeptides were eluted with 10% NH_4_OH. The eluted phosphopeptides were vacuum-lyophilized and desalted by C18 ZipTips (Millipore).

### Label-free liquid chromatography-tandem mass spectrometry

#### LC-MS/MS analysis

The tryptic peptides of 0.5 ug were dissolved in solvent A (0.1% formic acid in 2% acetonitrile) and then separated on a homemade reversed-phase analytical column of 25-cm length, 100 μm i.d. (packed with 1.9 μm/120 Å ReproSil-PurC18 resins) using NanoElute Ultra-Performance Liquid Chromatography (UPLC) system. The gradient was comprised of an increase from 7% to 24% over 72 min, 24% to 32% over 12 min, and climbing to 80% in 3 min, then holding at 80% for the last 3 min. All at a constant flow rate of 450 nL/min. The peptides separated by the UPLC system were then subjected to ionization by both electron spray (the Capillary) and captivespray source, followed by tandem mass spectrometry (MS/MS) in timsTOF Pro (Bruker Daltonics, USA) for analysis. The electrospray voltage applied was 1.6 kV. 1/k0 was set as 0.75-1.40 V·s/cm^2^; resolution was set as custom; ramp time was set as 100.0 ms; spectra rate was set as 9.43 Hz, and lock duty cycle was set to 100%. The m/z scan range was 100 to 1,700 for the MS2 spectrum. The data acquisition used a parallel accumulation serial fragmentation (PASEF) procedure. We selected precursors with charge states 0 to 5 for fragmentation, and 10 PASEF-MS/MS scans were acquired per cycle and set the dynamic exclusion time as 30 s. The overall procedures for phosphoproteomics were similar, except that the peptides were dissolved in solvent C (0.1% formic acid in water) and separated by an IonOpticks C18 reversed-phase analytical column (100 μm i.d. × 25 cm). The elution gradient was set as: 0–50 min, 2%~22%; 50–52 min, 22%~35%; 52–55 min, 35%~90%; 55–60 min, 90%. The flow rate was set constantly at 300 nL/min.

#### Database search

The resulting MS/MS data were processed using the Maxquant search engine (v.1.6.6.0) using “match between runs”, “second peptide search” and LFQ. Tandem mass spectra were searched against the human SwissProt database concatenated with reverse decoy database. Trypsin was specified as cleavage enzyme allowing up to 2 missing cleavages. The minimum length of peptide segment was set as 7 amino acid residues and the maximum modification number of the peptide segment was set to 5. The mass tolerance for precursor ions was set as 20 ppm in First search and 20 ppm in Main search, and the mass tolerance for fragment ions was set as 0.02 Da. Alkylation (cysteine) was specified as fixed modification, while oxidation (methionine), acetylation (protein N-term), desamidization (asparagine and glutamine) were specified as variable modifications. The FDRs of both protein identification and peptide spectrum matching (PSM) were set to 1%. The addition of phosphorylation (serine, threonine, and tyrosine) as modifications was used for phosphoproteomic data.

### Bioinformatics analysis

#### Statistical analysis

We used horizontally normalized LFQ (MaxQuant) as a quantitative protein intensity. This value is divided by the mean quantification of all samples and used as the normalized value for subsequent analysis. To compare the differences between these nine chordoma tumor tissues (CT) and their nine paired normal tissues (CN) that applied to proteiomics profiling, we compared the expression of each protein with paired *t*-test and determined differentially expressed proteins (DEPs) and differentially phosphorylation sites (DPSs) with |fold change (FC)| ≥ 2.0 and *p*-value < 0.05. We used statistical analysis methods including principal component analysis (PCA) and Pearson’s correlation coefficient to test sample repeatability. Statistical significance (*p* < 0.05) was assessed by using Student’s t-test. Based on clinical characteristics, we divided the tumor tissues into large (diameter > 5.0 cm, n=4) and small (diameter < 5.0 cm, n=5) (L/S) subgroups. DEPs and DPSs were determined similarly. The statistics were analyzed using ttest_ind function in scipy of Python 3.7.6.

#### Pathway enrichment analyses and hierarchical clustering

For the annotation and enrichment of DEPs, Kyoto Encyclopedia of Genes and Genomes (KEGG) database were classified against all identified proteins by two-sided Fisher’s exact probability test using fisher_exact function in scipy.stats of Python 3.7.6. The KEGG with an adjusted *p*-value < 0.05 was considered significant.

To find the functional correlation of different groups between DEPs or DPPs, we performed clustering analysis by hierarchical clustering and visualized it by heatmap based on p-values from Fisher’s exact test using pheatmap function in R package “pheatmap”. The horizontal dimension of the heatmap represented Fisher’s exact test results of different groups, while the longitudinal one described functional classification.

#### Protein-Protein Interaction (PPI) Network

The database accession numbers or protein sequences of all DEPs in different groups were searched in the STRING database (version 11) for protein-protein interactions. We fetched all interactions with a confidence score ≥ 0.7. Interaction network form STRING was visualized *via* forceNetwork function in R package “networkD3”.

#### Categorical gene set enrichment analysis (GSEA)

GSEA was performed on total proteomics and phosphoproteomics with GSEA v4.0.3 software (34). Official gene sets were downloaded from the GSEA website (www.broad.institute.org/gsea/) for enrichment. A permutation number of 1000 was adopted.

### Kinase activity prediction

#### Prediction of kinase-substrate regulations

We adopted iGPS V1.0 software (35) which utilized GPS2.0 algorithm for the prediction of site-specific kinase-substrate relations, and PPI information was used as the major contextual factor to filtrate potentially false-positive hits.

#### Prediction of kinase activities

We used Gene Set Enrichment Analysis (GSEA) method that adopted from the established PTM signature enrichment analysis (PTM-SEA) (36) to predict kinase activities. Normalized enrichment scores (NESs) of enrichment results were regarded as kinase activity scores. For each kinase, the kinase was predicted as positive if the predominant change of substrates was an increase in phosphorylation and vice versa.

#### Kinase-pathway network analysis (KiPNA)

We used a network analysis framework that is developed for relating kinase signaling with pathway dysregulation (37). Nodes stand for significantly enriched proteins, leading edge phosphosites, enriched kinases, and enriched pathways. The connected edges include enriched kinases, leading-edge phosphosites connect to target proteins, and proteins connect to enriched pathways. Networks were visualized using cytoscape.

### Cell line, cell culture, and transfection

Human chordoma cell lines JHC7 and U-CH1 were purchased from American Type Culture Collection (ATCC, Manassas, VA, USA). JHC7 cells were cultured in DMEM: F12 medium (ATCC^®^ 30-2006™, ATCC, USA) and U-CH1 cells were cultured in RPMI-1640 medium (11875093, Gibco, US) supplemented with an additional 1% L-glutamine (25030081, Gibco, US). To culture U-CH1, coating buffer (50 μg/mL rat tail type I collagen (354236, BD Biosciences) was added to the culture flask for one hour at room temperature prior to adding cells. All cells were cultured with 10% characterized fetal bovine serum (FBS) (10099141, Gibco, US), 10 units/mL penicillin and 10 mg/mL streptomycin (10378016, Gibco, US), and maintained in humidified incubators at 37°C with 5% CO_2_.

To knock down endogenous AURA, CDK9, and MOK, siRNA constructs were generated with the target sequences shown in **Table S4**. The siRNAs were purchased commercially (HanBio Inc., Shanghai, China). Chordoma cells were transfected with siRNA at a final concentration of 50 nM using Lipofectamine 3000 transfection reagent (Catalog No. L3000015, Invitrogen, USA). The suppression efficiency of AURA, CDK9, and MOK was analyzed by western blot on the third day post transfection.

### Western blotting

Proteins were lysed and extracted by RIPA (Thermo Fisher Scientific) cell lysis buffer supplemented with protease inhibitor and phosphatase inhibitor cocktails. Protein quantification was measured by the Pierce BCA protein assay kit (Thermo Fisher Scientific). 10 ug of total proteins were applied to SDS-PAGE electrophoresis, transferred to PVDF membrane, and detected by conventional protocols for western blotting. Primary antibodies against Aurora kinase (AURA) (66757-1-Ig, Proteintech, US), Cyclin-dependent kinase 9 (CDK9) (2316, CST, US) and MAPK/MAK/MRK overlapping kinase (MOK) (23926-1-AP, Proteintech, US), Brachyury-T (81694, CST, US) and β-actin (4970, CST, US), and subsequently with the appropriate horseradish peroxidase–conjugated secondary antibodies (7074, CST, US).

### Cell proliferation analysis

Cell proliferation was measured by a Counting Kit-8 (CCK-8) detection kit (Catalog No. CK04, Dojindo Molecular Technologies, Japan). The cells were seeded in a 96-well plate with 3000 cells per well and treated with siRNA transfection. At the indicated time points, 10 μL of CCK-8 solution was added, followed by incubation at 37°C for two hours, and absorbance at 450 nm was determined.

For *in vitro* inhibitor assays, AZD4573 (S8719, Selleck) was dissolved to 10 mM in DMSO and then dosed to yield a final DMSO concentration of ≤ 0.3%. Chordoma cells was treated with different concentration of AZD4573 from 0.4 nM to 4000 nM (ten-folds dilution) for nine days and the cell proliferation was tested by CCK-8 as well.

### Immunohistochemical and hematoxylin-eosin staining

IHC and HE staining were performed on formalin-fixed, paraffin-embedded tissues as previously described (38). Briefly, samples were incubated with primary anti-Brachyury antibody (81694, CST, USA, 1:200 dilution) at 37°C for 1 hour and second antibody at 37°C for 30 min. Then, the samples were treated with the DAB Substrate kit (PV-8000, ZSGB-BIO, China).

## Results

### Overview of the global proteomic and phosphoproteomic landscape with respect to chordoma

**Figure 1:**
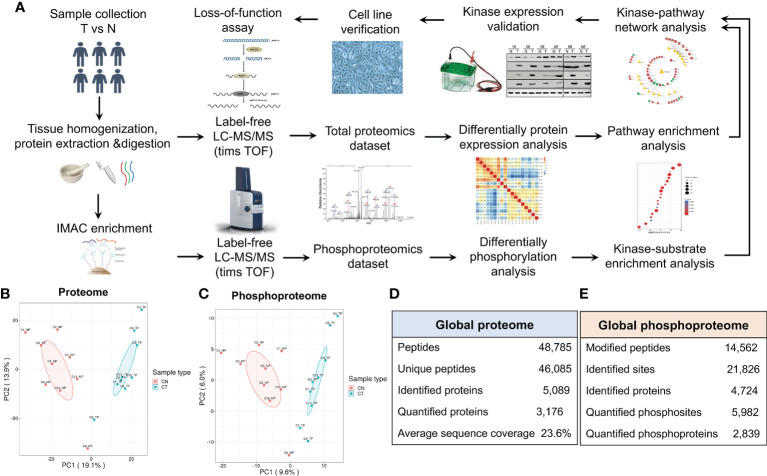
Characterization of the proteome and phosphoproteome in chordoma tumor and normal tissues. **(A)** General pipeline of (phospho)peptide enrichment and the quantitative mass spectrometric protocol followed by pathway analyses and biochemical validation. **(B, C)** Principal component analysis (PCA) of the quantified proteome **(B)** and quantified phosphoproteome **(C)**. **(D, E)** Statistical analysis of the 4D label-free proteomic **(D)** and phosphoproteomic **(E)** datasets.

We enrolled 15 patients who were diagnosed with chordoma according to both preoperative images and IHC examination (**Table 1**; **Figures S1A–D**): samples from nine of these were subjected to proteomic study and the other six were used for western blot analysis. Since it is impracticable to sample the embryonic notochord (the ideal chordoma control), we compared CT to their CN as performed by many other investigative groups (32, 39, 40) (our entire protocol is illustrated in **Figure 1A**). We then chose to further validate some intriguing and significant targets retrieved from the high-throughput LC-MS/MS results.

PCA of both the proteome and phosphoproteome revealed similarities among the sample groups (**Figures 1B** **, C**), confirming the validity of our dataset and indicating its reliability for further assessment. We then added mobility (4-D label-free) to our LC-MS/MS analysis to increase the peptide-identification depth. In total, we identified 46,085 unique peptides from 5,089 proteins and 21,826 phosphosites from 4,724 proteins (**Figures 1D** **, E**).

### Identification of differentially expressed proteins and alterations in the Wnt-signaling pathway

**Figure 2:**
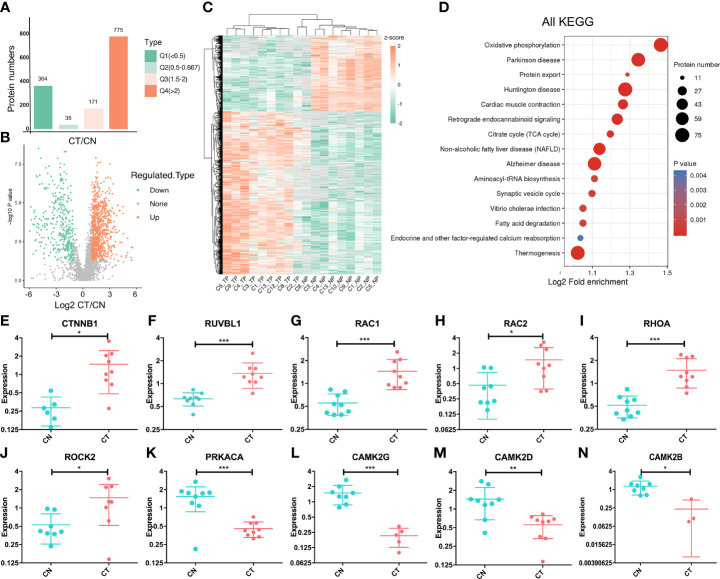
Targeted proteomic study and altered expression of Wnt-signaling-related proteins. **(A)** Statistical comparison of regulated proteins between chordoma tissues (CT) and their paired normal adjacent muscle tissues (CN) by Q category. Q1, 0 < ratio ≤ 0.5, corresponding to downregulated proteins; Q2, 0.5 < ratio ≤ 0.667; Q3, 1.5 < ratio ≤ 2; Q4, ratio >2, corresponding to upregulated proteins. Protein numbers for Q1-Q4 are 364/35/171/775, respectively. **(B)** Volcano plot of the distributional patterns of statistical significance (-log P value) and magnitude of the changes (log_2_FC) for all DEPs. **(C)** Unsupervised hierarchical clustering heatmap of the significantly regulated proteins identified from chordoma tissues. Unique proteins (n=1,139; rows) were significantly identified from nine paired samples (columns). TP, tumor proteins; NP, normal proteins. Unsupervised hierarchical clustering was performed using the Cluster program with Pearson correlation and pairwise complete-linkage analyses. **(D)** KEGG pathway-enrichment analysis was executed to identify important pathways that depended upon all DEPs. The colored blocks that correspond to functional classification indicate the magnitude of enrichment, and are displayed by colors ranging from blue (weak enrichment) to red (strong enrichment). **(E–N)** Relative normalized expression of CTNNB1 **(E)**, RUVBL1 **(F)**, RAC1 **(G)**, RAC2 **(H)**, RHOA **(I)**, ROCK2 **(J)**, PRKACA **(K)**, CAMK2G **(L)**, CAMK2D **(M)**, and CAMK2B **(N)**. The vertical axis signifies log (2). CN, blue; CT, red. Student’s paired t-test was applied to distinguish the expression differences; *, p < 0.05, **, p < 0.01, ***, p < 0.001.

We divided the DEPs into four quantified groups (Q1–Q4) according to their differentially expressed magnification and determined that 364 proteins in Q1 (0 < ratio ≤ 0.5) and 775 proteins in Q4 (ratio > 2) were down- and up-regulated proteins, respectively (**Figure 2A**). These 1,139 proteins were regarded as significantly DEPs, and visualization of their distribution patterns indicated that a majority were upregulated (**Figure 2B**). Hierarchical clustering of DEPs indicated that the protein expression status of CT was different from that of CN **(** **Figure 2C**). Then we adopted functional KEGG-pathway analysis on all the DEPs to evaluate their biological processes, and noted that oxidative phosphorylation (the metabolic pathway linking electron transport and phosphorylation) was dramatically altered in chordoma tissues (**Figure 2D**). Since most of the proteins were over-expressed in chordoma, we then analyzed the KEGG pathway for the upregulated proteins and ascertained that non-homologous end-joining was dramatically enriched (**Figure S2A**). We also executed GSEA to better elucidate the functions of the DEPs (**Figure S2B**) and found oxidative phosphorylation and lysosome to be enriched using both KEGG and GSEA (**Figures S3A–D**), suggesting their relatively robust functions in chordomagenesis.

As has been stated previously, the Wnt-signaling pathway plays a crucial role in embryonic and notochord development. We therefore assessed whether this signaling pathway was aberrantly regulated in chordoma, we determined that at least 10 proteins of the ~100 known proteins in the Wnt-signaling pathway exhibited anomalies (six were upregulated and four were downregulated; **Figures 2E** **–N**). The pivotal protein β-catenin (gene symbol: *CTNNB1*) showed an elevated (~two-fold) expression (**Figure 2E**), and as a subunit of the cadherin protein complex that acts as an intracellular signal transducer, mutations and increased expression of β-catenin are associated with many cancers (41). Our findings signified that chordomas shared similar behaviors. For example, calcium/calmodulin-dependent protein kinase type II (CaM-kinase II) is a ubiquitous Ser/Thr-directed protein kinase comprising a family of four genes (alpha, beta, gamma, and delta) that regulates the Wnt pathway (42). In our dataset, three of these four proteins (CAMK2G, CAMK2B, and CAMK2D) were downregulated (**Figures 2L** **–N**), suggesting a potential for aberrant regulation of CaM-kinase II and its corresponding phosphorylation.

### Integration of the proteome and phosphoproteome reveals multiple regulatory levels

**Figure 3:**
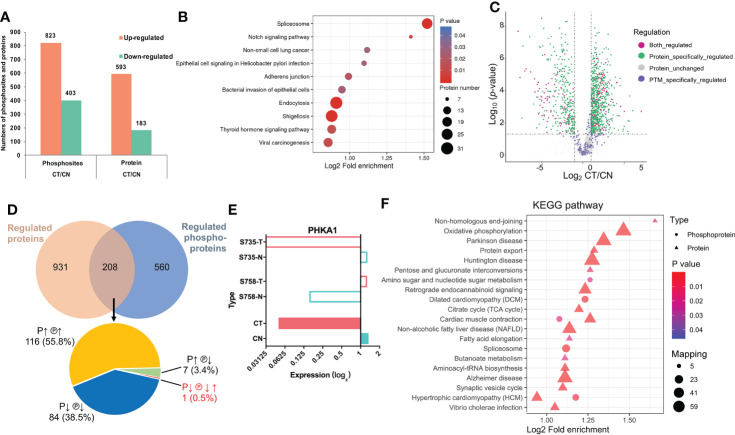
Phosphoproteomic analysis and its combinative interpretation in relation to the proteome. **(A)** Statistical comparison of differentially phosphorylated sites and proteins between CT and CN. **(B)** KEGG pathway-enrichment analysis was implemented separately for the upregulated DPPs. **(C)** Volcano plot of the distribution patterns of protein-specific (green), phosphorylation-specific (purple), and combined (magenta) protein regulations. **(D)** Venn diagram of proteomics and phosphoproteomics analyses. Two-hundred eight proteins were found to be dually regulated: of these, 116 (55.8%) were both over-expressed (P↑) and hyper-phosphorylated (℗↑); 84 (38.5%) exhibited the opposite conditions (P↓ ℗↓); seven (3.4%) proteins were over-expressed but hypo-phosphorylated (P↑ ℗↓); and while the expression of one (0.5%) protein was downregulated (P↓), it possessed two phosphosites that showed opposing patterns (℗↓↑). **(E)** Detailed information with respect to PHKA1 regulation. Although serine735 (S735) and serine758 (S758) were phosphorylated in opposing fashions, their expression was dramatically downregulated. Colors are the same as in **Figures 2E–N** (CN, blue; CT, red). **(F)** Combined KEGG pathway-enrichment analysis for DEPs and DPPs. Circles and triangles designate DEPs and DPPs respectively, with different colors denoting their differential expression (red represents upregulation and blue represents downregulation).

In addition to determining protein expression levels, we quantified the DPPs to elucidate their potential impacts, and identified 823 upregulated phosphosites that corresponded to 593 proteins and 403 downregulated phosphosites that corresponded to 183 proteins (**Figure 3A**). We subsequently implemented KEGG pathway enrichment analysis for up- and down-regulated DPPs, demonstrating that 34 pathways were enriched, and we delineated the top 10 upregulated pathways in **Figure 3B**. Notably, spliceosome (involving 23 DPPs) was the most significantly different between CT and CN tissues (**Table S1**), and five proteins that contained multiple phosphorylation sites were dually phosphorylated and functioned in both up- and down-regulation (**Table 2**), thereby indicating a complicated regulatory function for protein PTM. The Notch-signaling pathway was also markedly enriched, indicating its aberrant regulation in chordoma formation (**Figure 3B**).

**Table 2:** Proteins with both up- and down- regulated phosphorylated sites.

Protein accession	Name	Position	Ratio	P value	Regulated Type
O14639	ABLIM1	490S	0.333	0.049	Down
		655S	7.651	0.021	Up
		706S	4.732	0.009	Up
P14618	PKM	77S	0.166	0.000	Down
		127S	0.226	0.006	Down
		519S	2.400	0.008	Up
P27816	MAP4	696S	0.361	0.032	Down
		928S	7.811	0.027	Up
Q15149	PLEC	201S	0.225	0.000	Down
		1435S	0.266	0.008	Down
		2782S	7.928	0.045	Up
		4618S	10.853	0.014	Up
		4385S	13.973	0.007	Up
Q8WWI1	LMO7	704S	2.541	0.015	Up
		805S	0.357	0.010	Down
		991S	0.424	0.022	Down
		1510S	0.312	0.004	Down
		1586S	0.294	0.008	Down

We next analyzed the proteins that were regulated at both the expression and post-translational modification (PTM) levels (**Figures 3C** **, D**). Using the intersection of the proteomic and phosphoproteomic datasets, we uncovered 208 proteins of which 55.8% were upregulated at both levels (**Figure 3D**, **Table S2**). Intriguingly, the phosphorylase b kinase regulatory subunit alpha (PHKA1) displayed a unique pattern, with diminished protein abundance but containing both hyper- and hypo-phosphorylation sites (**Figure 3E**). We also noted seven proteins that showed downregulated phosphorylation but upregulated expression, indicating that phosphorylation influenced their protein abundance and consequent functions. KEGG pathway-enrichment analysis was ultimately performed on the 208 proteins to characterize their possible biologic functions more intuitively (**Figure 3F**).

### Cell proliferation-related kinases promote chordomagenesis

**Figure 4:**
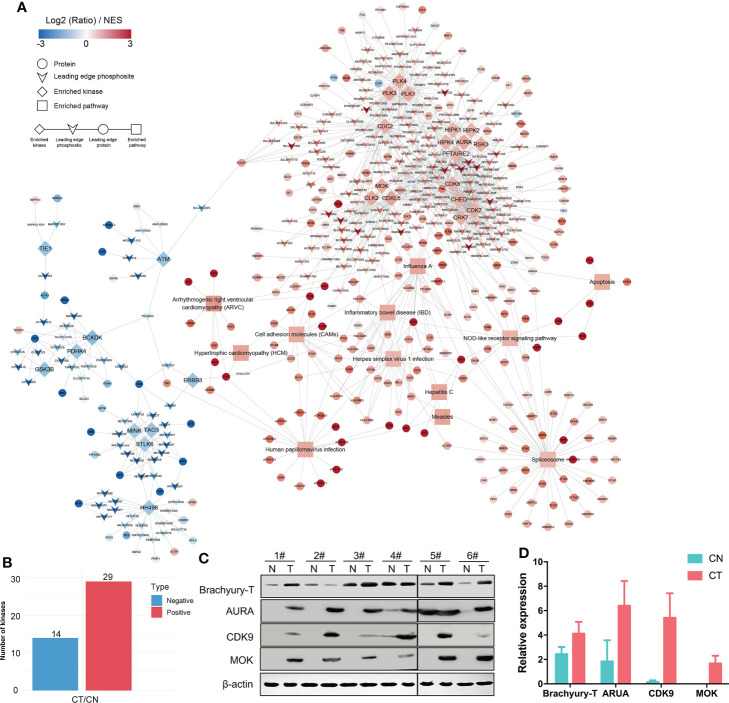
KiPNA analysis and the expression validation of specific proteins. **(A)** Kinase-pathway network analysis (KiPNA) indicating the proteomic and phosphoproteomic signaling network in chordoma. **(B)** Fourteen and 29 kinases were conjectured to be negatively and positively regulated, respectively. **(C)** The elevated expression of brachyury-T, AURA, MOK, and CDK9 were verified by western immunoblotting analysis. The samples were derived from six independent patients who were distinct from the patients whose samples were used for mass spectrometry. T, tumor tissues; N, normal tissues. **(D)** Densitometric quantification of western blot results from **(B)** and presented relative to β-actin expression. The data represent the mean ± SD of each experiment carried out in triplicate.

To investigate our datasets more systematically and to uncover some potential targets, we executed KiPNA tailored for phosphoproteomic profiles (**Figure 4A**). KiPNA is an integrated computational approach that enables us to predict the activities of kinases based on the differences in their substrate phosphorylation. We identified 28 possible kinases with altered activity (**Figure 4B** **;** **Table S3**), and initially chose the top 10 for validation (data not shown). Three of these kinases (Aurora kinase A, AURA; Cyclin-dependent kinase 9, CDK9; and MAPK/MAK/MRK overlapping kinase, MOK) appeared to show significant differences between CT and CN samples. Aurora kinases are essential enzymes in the control of the cell cycle. For instance, AURA is critical to the regulation of cancer progression, and its mutations and deregulations are associated with several cancers (43). CDK9 reactivates epigenetically silenced genes in cancer, and inhibition of CDK9 by drugs such as flavopiridol, dinaciclib, seliciclib, SNS-032, and RGB-286638 is exploited in cancer therapy (44, 45). MOK is closely related to serine/threonine protein kinases in the protein kinome, and the expression of MOK is augmented in various tumors (46). Considering these results, we analyzed the roles of these kinases in chordoma.

**Figure 5:**
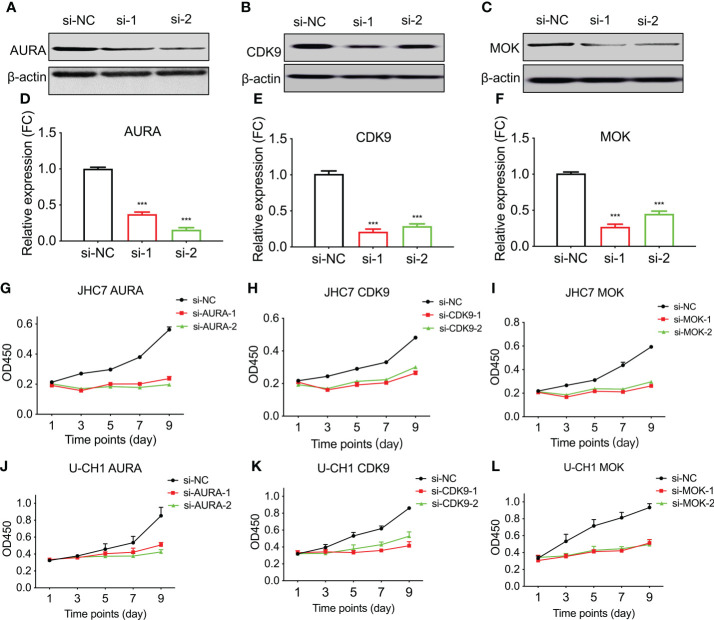
Knockdown of AURA, MOK, and CDK9 compromises chordoma cell line proliferation. The human chordoma cell lines JHC7 and U-CH1 were used to evaluate the proliferative effects of kinases. **(A–C)** The efficiency of siRNA-knockdown transfection was confirmed by western blotting of whole-cell extracts from JHC7 cells for AURA **(A)**, CDK9 **(B)**, and MOK **(C)**. si-NC, negative control; si-1 and si-2, two independent siRNAs. **(D–F)** Densitometric quantification of western blot results from **(A–C)**. Student’s t-test was used. **(G–L)** Cellular proliferation after siRNA transfection was quantified by CCK8 assays. Cell growth of JHC7 **(G–I)** and U-CH1cell lines **(J–L)** was significantly attenuated by knockdown of AURA, CDK9, and MOK. The data were acquired from three biologically and independently repeated experiments (n=3, data are mean ± s.e.m.) ***: P< 0.001.

We first confirmed that the specific marker of chordoma, brachyury (gene symbol: *TBXT*), was over-expressed in chordoma tissues when compared with normal tissues in six different cases (**Figure 4C**, **Table 1**). We then compared and validated the elevated expression of these kinases in CT to CN [although a few special cases were inconsistent, most likely due to individual heterogeneity (**Figures 4C** **, D**)]. Second, to evaluate the roles of AURA, CDK9, and MOK in chordoma cell proliferation, we silenced AURA, CDK9, and MOK protein expression in chordoma cell lines using siRNAs, and demonstrated that knockdown efficacy to be over 70% in JHC7 cells (**Figures 5A** **–F**). Despite the slow growth of chordoma cells, JHC7 proliferation rate after knockdown of AURA, CDK9, and MOK was nevertheless significantly suppressed relative to control cells as measured by CCK8 assay (**Figures 5G** **–I**). We observed similar results with U-CH1, another chordoma cell line (**Figures 5J** **–L**). These results collectively indicate that AURA, CDK9, and MOK promote chordoma oncogenesis and could therefore be promoted as potential therapeutic targets in the treatment of chordoma.

### Inhibition of CDK9 reduces tumor growth in chordoma

Since numerous kinase inhibitors have been discovered and developed, we assessed whether their inhibition would also lead to consequences analogous to those with siRNA transfection. Aurora family members are crucial to faithful mitotic transition, and their specific inhibitors include Barasertib (AZD1152) and Alisertib (MLN8237) (47). There also exists an abundance of CDK9 inhibitors, from the first-generation inhibitors Alvocidib (flavopiridol) and Seliciclib (roscovitine/CYC202) to the more specific and more potent second-generation inhibitors as represented by BAY1143572 (atuveciclib) and AZD4573 (45). There are, however, far fewer data with respect to inhibitors of MOK. Considering their inhibitory potency and selectivity, we chose CDK9 and AZD4573 for further validation.

**Figure 6:**
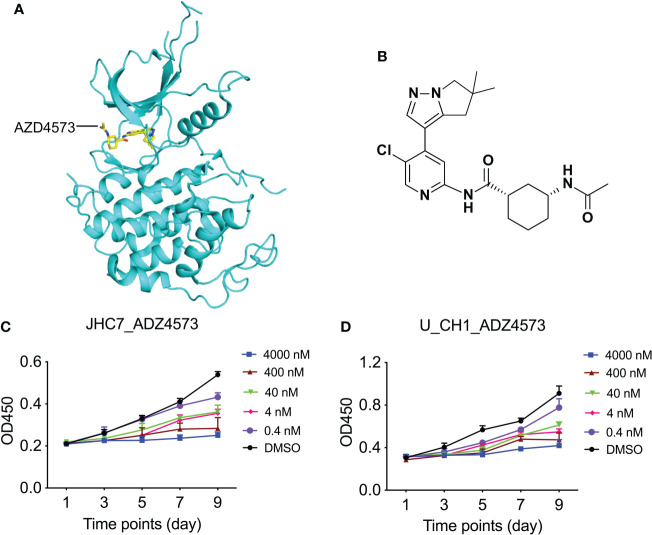
Dose-dependent treatment of AZD4573 results in cellular proliferation in chordoma cell lines. **(A)** Structural cartoon representation of CDK9 protein (pdb ID: 6Z45); AZD4573 is depicted as yellow sticks. **(B)** Chemical structure of AZD4573. **(C, D)** AZD4573 drug-sensitivity assays. JHC7 **(C)** and U-CH1 **(D)** cells were treated with graded concentrations of AZD4573 (from 0.4 nM to 4000 nM) and proliferation was subsequently determined with the CCK-8 cell-proliferation assay.

CDK9 is a functional subunit of P-TEFb (positive transcription elongation factor) and regulates transcriptional pause-release during gene transcriptional elongation (48). AZD4573 was optimized as a highly selective CDK9 inhibitor with an IC50 of less than 4 nM and exhibited a greater than 10-fold selectivity for CDK9 over CDKs 1–7 (**Figures 6A** **, B**); it is currently regarded as a clinical candidate for the treatment of hematologic malignancies (49, 50). We treated chordoma cells with various concentrations (using 10-fold dilutions) of AZD4573 and found that, commensurate with increasing concentrations of AZD4573, the inhibitory effects on cellular proliferation were also gradually augmented (**Figures 6C** **, D**). These results indicate that functional inhibition of a key kinase suppresses chordoma cell proliferation and may thus provide a therapeutic option for the treatment of chordoma.

### Chordoma tumor size is correlated with cytoskeletal anomalies

**Figure 7:**
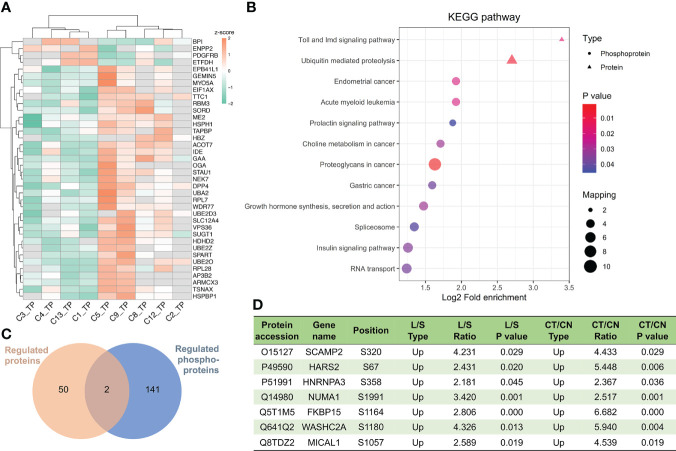
Important proteins and pathways related to chordoma tumor size. **(A)** Unsupervised hierarchal clustering disclosed differential protein regulation between large and small chordomas. C1/C3/C4/C13 patients belonged to the large (L) group and C2/C5/CC8/C9/C12 to the small (S) group. **(B)** Combined KEGG pathway-enrichment analyses for both DEPs and DPPs are depicted as bubble charts. **(C)** Venn diagram of the proteomics and phosphoproteomics analyses for comparisons between L and S. **(D)** Seven proteins whose phosphorylation levels were augmented in both CT/CN and L/S groups.

Due to the slow proliferation rate of chordomas and their adjacency to the spine, chordoma tumor size is directly related to patient survival rate (51). We regarded tumor size to be large if its maximal diameter was greater than 5.0 cm, and small if under 5.0 cm, and thereby noted four large (L, n=4) and five small (S, n=5) chordomas (**Table 1**). Unsupervised hierarchal clustering of these samples indicated differential protein expression patterns (**Figure 7A**), while Toll- and Imd-signaling pathways and ubiquitin-mediated proteolysis were significant functional KEGG enrichments with respect to both DEPs and DPPs (**Figure 7B**). Only two proteins were regulated at both the expression and phosphorylation levels (**Figure 7C**). To ascertain which proteins were uniformly altered in the CT/CN and L/S groups, we compared these two datasets and uncovered seven proteins in which phosphorylation levels were augmented in both (**Figure 7D**). We hypothesize that these dually changed proteins are plausible in promoting chordoma cell proliferation and tumor growth.

## Discussion

Chordomas are dual epithelial-mesenchymal tissue tumors that show slow progression, but despite their low malignancy, chordomas always manifest a high recurrence rate and frequently lead to local invasion and distant metastasis in advanced stages (52, 53). Chordoma is resistant to conventional chemotherapy and radiotherapy and surgical eradication remains challenging due to the complicated anatomical location of the tumors; patients are also vulnerable to relapse. It is therefore of the utmost necessity to discern the detailed molecular underpinnings of chordoma progression, and the exploration of novel therapeutic targets would also be of great value for chordoma patients.

Several investigators have assessed the genomic stability, epigenetic aberrations, genetic variations, gene transcription, microRNA expression profiles (23–28), and proteomic profiles of chordomas (32, 54). However, ours is the first-ever phosphoproteomics analysis of chordoma. We herein illuminated the PTM landscape together with protein expression by executing high-throughput, 4D, label-free proteomic and phosphoproteomic analyses, and then combined the bioinformatic results with cellular and biochemical verification so as to elucidate potential phosphorylative regulatory mechanisms in chordomas. The ideal control would be normal non-tumoral notochordal tissues, but as normal notochord is small and limited to the embryonic stage, sufficient material needed for experimentation is too difficult to procure; thus, as for numerous other studies (32, 39, 40), we utilized normal adjacent muscle tissues as a substitute control as both tissues types originate from mesenchymal cells.

From nine paired tissues, we identified 5,089 proteins and 4,724 phosphorylated proteins. The key regulators of Wnt signaling are critical to embryonic development, and we noted marked alterations in CT tissues relative to CN tissues (**Figure 2**). Using intersection analysis, we identified 208 proteins that were dually regulated, and observed alterations in multiple pathways at both proteomic and phosphoproteomic levels (**Figure 3**). Moreover, we uncovered 28 kinases as possible therapeutic targets through KiPNA, of which AURA, CDK9, and MOK were validated both *in vivo* and *in vitro* (**Figures 4** **and** **5** **)**, and their inhibition by small molecules proved their potential as drug targets (**Figure 6**). Considered the tumor size, we determined that secretory carrier-associated membrane protein 2 (SCAMP2) and the other seven proteins were upregulated in both the CT/CN and Large/Small groups (**Figure 7**). These data provided a basis for the aforementioned phosphorylated proteins being related to malignancy *via* the promotion of tumor-cell amplification.

Small molecules that specifically target brachyury-T have been screened (55), and several kinase inhibitors have also been evaluated as to their suppression of chordoma tumor cell growth (29, 30). However, these few relevant publications were limited to assessments of phosphorylation and specific kinases in chordomas, while our datasets provided more suitable and more widely applicable kinase candidates for future research.

Although we acknowledge limitations to the present study, such as the relatively small sample size (n=9 for our proteomics analysis) due to the rarity of chordomas and the lack of *in vivo* validation models such as xenografts, our findings still reveal useful information regarding chordomas. Our proteomic and phosphoproteomic data provide much-needed options for additional studies with respect to their validation and the application and establishment of more efficient therapies for chordoma treatment.

## Data availability statement

The datasets presented in this study can be found in online repositories. The names of the repository/repositories and accession number(s) can be found in the article/**Supplementary Material**.

## Ethics statement

The studies involving human participants were reviewed and approved by the ethical review board of the Peking University Third Hospital [M2019233]. The patients/participants provided their written informed consent to participate in this study.

## Author contributions

This work was performed by all authors. JH, HO and FW performed the majority of experiments. HO and QZ executed the experiments. JH and WY performed the majority of data and statistical analysis. ZL and LJ conceived and designed experiments. JH, HO and WY wrote and edited the manuscript. ZL, LJ and WY directed the study. FW collected chordoma tissues and clinical patient information. All authors contributed to the article and approved the submitted version.

## Funding

This work was supported by the National Natural Science Foundation of the P. R. of China (82071658), Beijing Natural Science Foundation (7204327), the Beijing Nova Program (Z201100006820010), Capital’s Funds for Health Improvement and Research (2020-4-40916), the Key Clinical Program of Peking University Third Hospital (BYSY2017002), and Clinical Medicine Plus X - Young Scholars Project, Peking University, the Fundamental Research Funds for the Central Universities (PKU2021LCXQ005).

## Conflict of interest

The authors declare that the research was conducted in the absence of any commercial or financial relationships that could be construed as a potential conflict of interest.

## Publisher’s note

All claims expressed in this article are solely those of the authors and do not necessarily represent those of their affiliated organizations, or those of the publisher, the editors and the reviewers. Any product that may be evaluated in this article, or claim that may be made by its manufacturer, is not guaranteed or endorsed by the publisher.
