# Supplementary material for: Proteomics and phosphoproteomics of chordoma biopsies reveal alterations in multiple pathways and aberrant kinases activities
Source: Front Oncol. 2022 Sep 30;12:941046. doi: 10.3389/fonc.2022.941046 (PMC9563620; doi:10.3389/fonc.2022.941046)
Supplement: Supplementary file 1 [file DataSheet_1.docx]

**
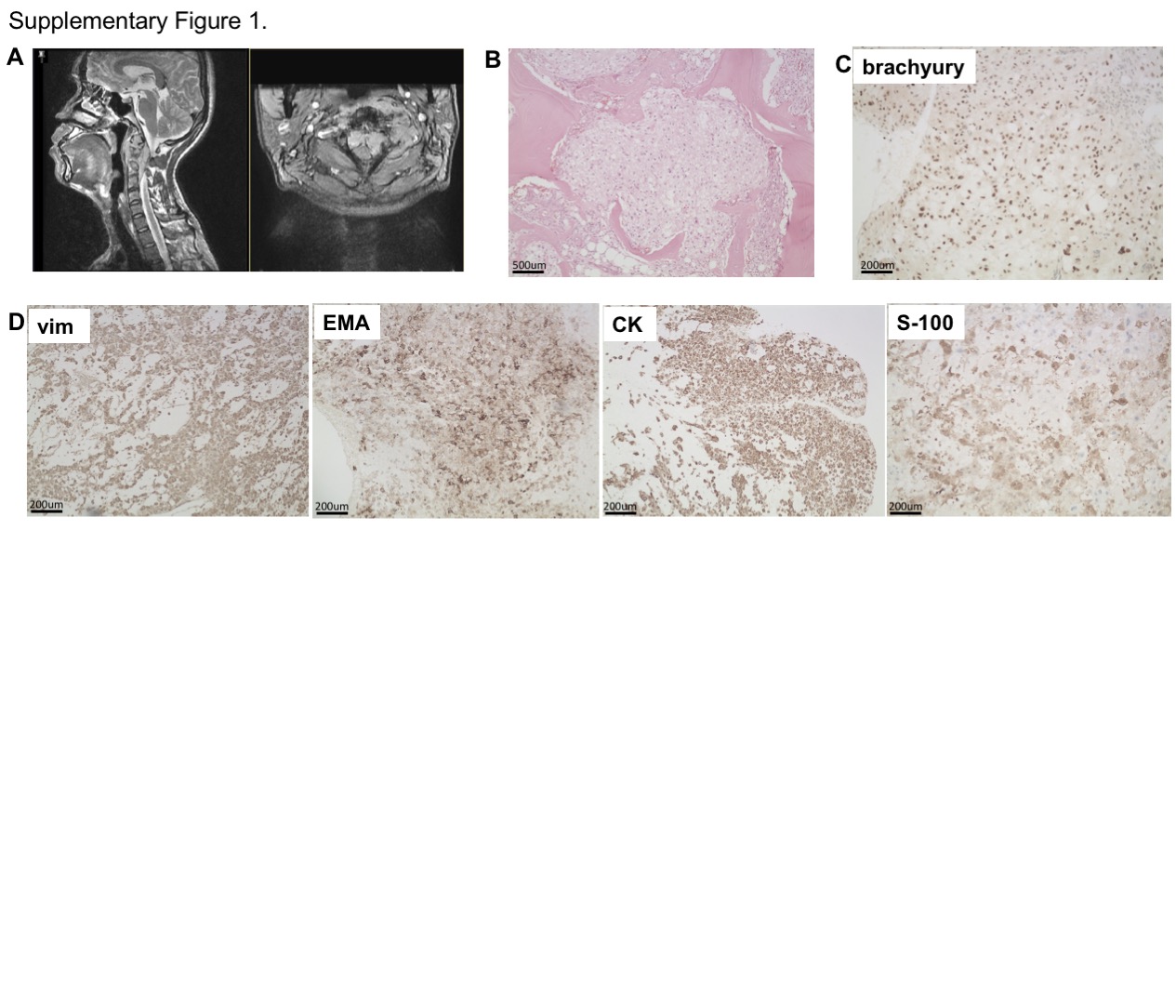
**

**Figure S1 Chordoma with different diagnose parameters.** (**A)** The sagittal and axial MRI images of cervical spine. Chordoma tumor has occupied the C1-C2 vertebrae bodies and compressed the spinal cord. **(B)** The morphological structure of chordoma with HE staining. **(C)** Immunohistochemistry staining for chordoma marker brachyury. **(D)** Immunohistochemistry staining for other Vimentin (vim), Mucin-1 (EMA), Casein kinase II (CK) and Protein S100 (S-100).


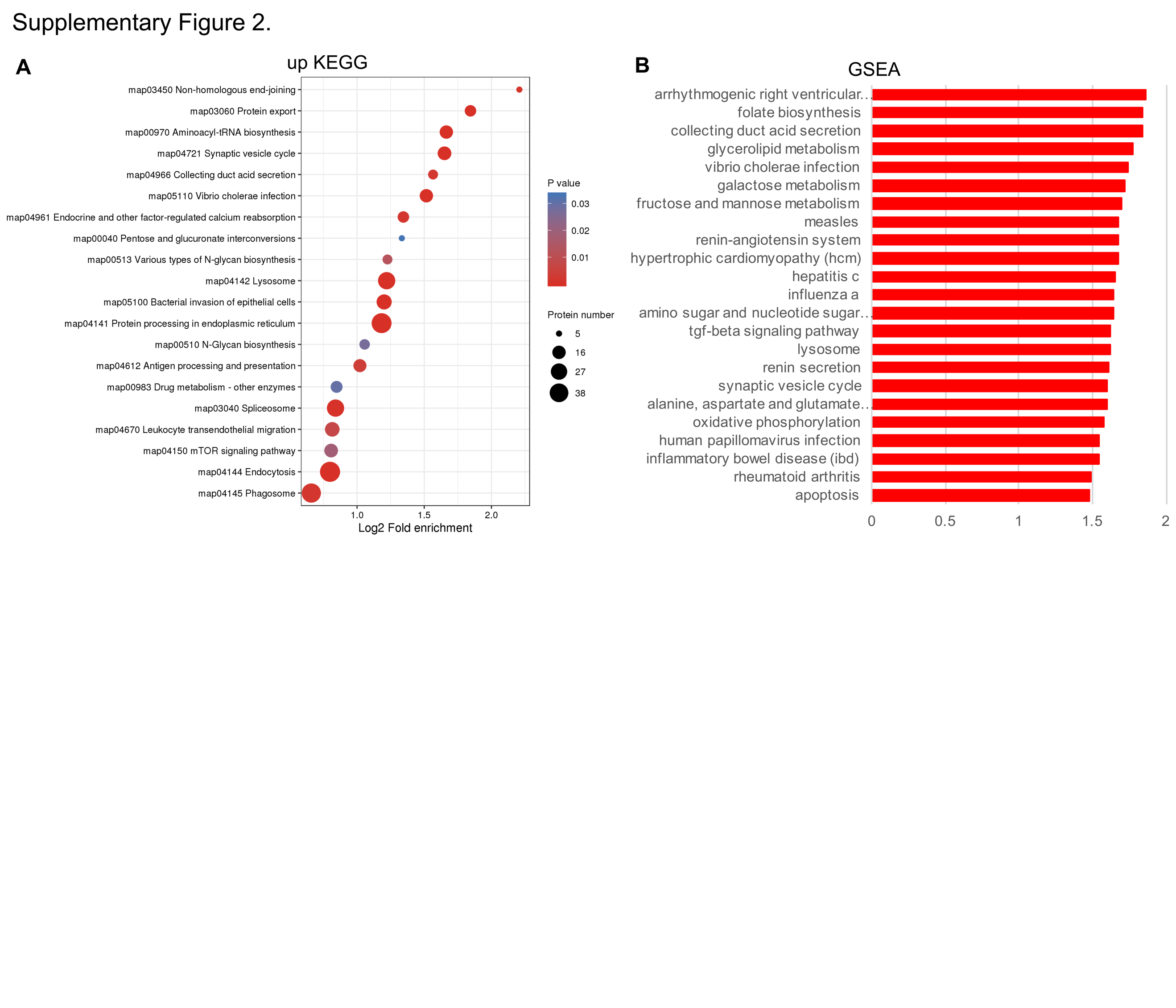


**Figure S2 Pathway enrichment analysis of DEPs. (A)** Schematics of KEGG enrichment for up-regulated proteins. (**B**) GSEA enrichment analysis for both up- and down-regulated DEPs.


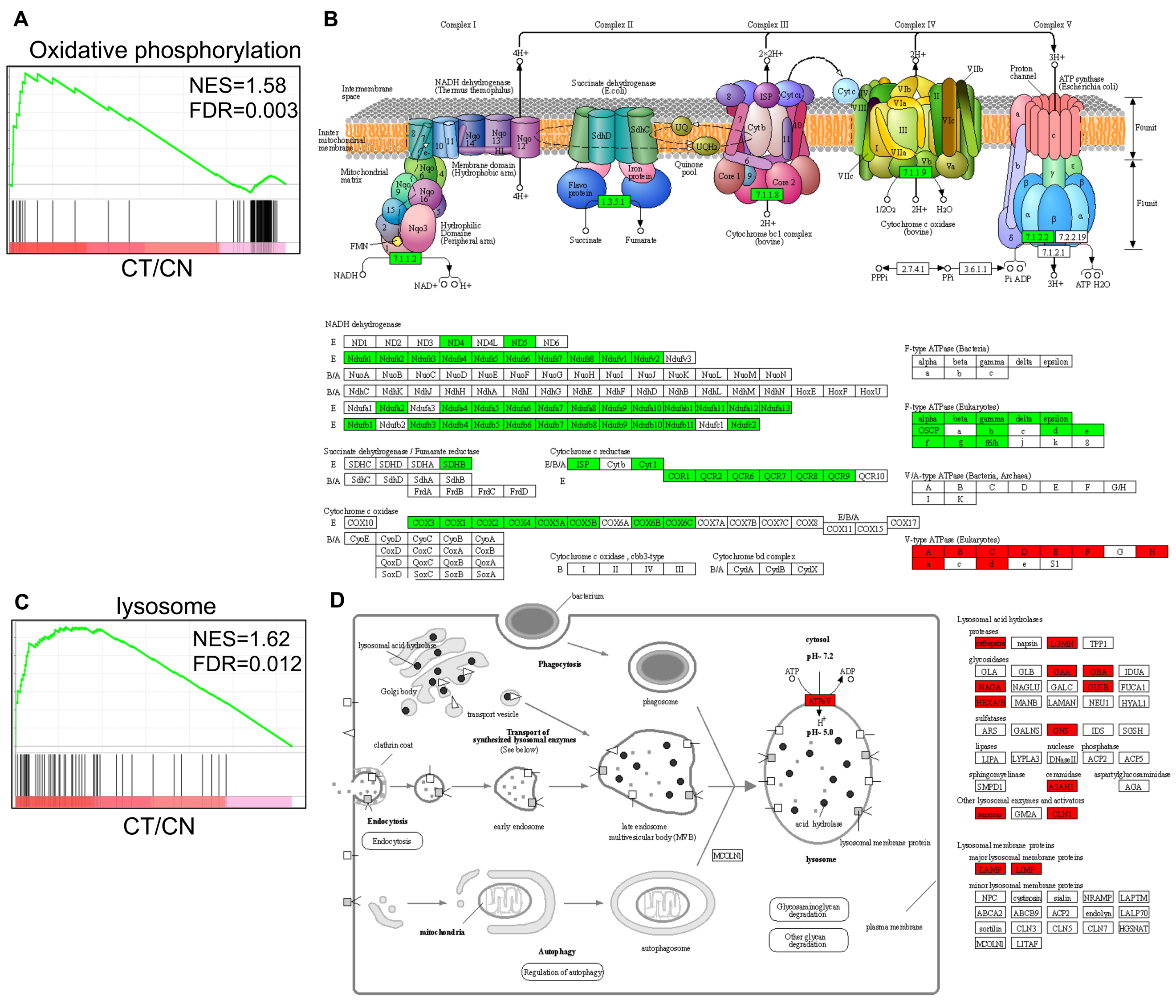


**Figure S3** **Oxidative phosphorylation and lysosome were two pathways that were regulated by both KEGG and GSEA methods.** (**A**) GSEA enrichment score for oxidative phosphorylation. NES, normalized enrichment score. (**B**) Differentially expressed proteins in oxidative phosphorylation. Red and green indicate up-regulated and down-regulated, respectively. (**C**) GSEA enrichment score for lysosome. (**D**) Differentially expressed proteins in lysosome illustrated with the same color in B.
